# The implications of complexity, systems thinking and philosophy for pediatricians

**DOI:** 10.1186/s13052-021-01031-6

**Published:** 2021-03-25

**Authors:** Jochen Ehrich, Jürgen Manemann, Velibor Tasic, Natale Gaspare DeSanto

**Affiliations:** 1 Children’s Hospital, Hannover Medical School, Carl-Neuberg-Straße 1, 30625 Hannover, Germany; 2 Hanover Research Institute for Philosophy, Hannover, Germany; 3 University Children’s Hospital, Medical School, Skopje, North Macedonia; 4 University of Campania Luigi Vanvitelli, Naples, Italy

**Keywords:** Children, Pediatrics, Complexity, Systems thinking, Philosophy, Salutogenesis

## Abstract

National service systems in child healthcare are characterized by diversity and complexity. Primary, secondary, tertiary and quaternary healthcare services create complex networks covering pediatric subspecialties, psychology, sociology, economics and politics. Can pediatrics exist without philosophy? Does the absence of integrating philosophical perspectives during conceptualization of pediatric care contribute to deficiencies in the service systems structuring child healthcare? Philosophy offers new ways of complex systems thinking in scientific and clinical pediatrics. Philosophy could improve coping strategies on different levels when dealing with ethics of research projects, individual child healthcare and crises of healthcare service systems. Boundary and ultimate situations experienced by severely sick children require help, hope and resilience. Patients and families as well as pediatricians and other caregivers must act in concert. All of them may benefit from consulting with philosophers. The aim of this article is to point out the risks of a strict separation of scientific insight and sensory experience affecting child healthcare in our modern society, which is dominated by technology, competition and lack of equity and time.

## Introduction

What is clinical philosophy and how could it lead to complex system thinking in pediatrics in order to cope with challenges and crises of children with severe diseases? Philosophy differs from psychotherapy in that it is not a therapy. Nonetheless, any philosophy that attributes a fundamental dimension to its potential practical relevance can be considered therapeutic [1]. This therapeutic dimension of philosophy can refer both to individual and general healthcare in the community. Socrates criticized both the individual who does not care for himself and human communities that are not properly set up, and he described the parallels between bad individual mental constitutions and a bad constitution of the state [1]. In this sense, there is a two-dimensional improvement of life: people only get better if the society in which they live gets better, and a society only gets better if the people in it become better people [1].

There are four different types of philosophers who could become involved in medicine. Philosophical pastoral care as a hermeneutic of existence can help patients to better understand their life and to find a viable way during moments of illness when they feel caught in an impasse. Clinical philosophy sees itself as an applied philosophy that “works as a medicine” [1]. From a historical perspective the strong connection between science and philosophy existed until the eighteenth century, however, this alliance broke thereafter [2]. A science such as medicine that deals with the mystery of life cannot be complete if it deals only with natural sciences. Healthcare services including pediatrics are part of complex institutional systems [3]. This is where the concept of philosophical practice comes in.

## Pediatrics as part of holistic healthcare service systems

Pediatrics and its high-tech care subspecialties like pediatric nephrology and its fractals like dialysis or transplantation are partial disciplines of complex and holistic medicine. De Santo [4] wrote: “The potential of complexity is explored along with new techniques and a wider use of artificial intelligence, as well as the links with philosophy, systems biology, systems medicine and systems pharmacology”. Henry Barnett wrote in “philosophy and ethics of multi-center international controlled clinical trials in children” that this subject had evoked intense reactions: “I believe it is because it is such a clear example of a more general conceptual difference between opinions and conclusions drawn from general experience and non-systematic observations and those based on scientific principles and logical reasoning. In medicine the conflict is especially sharp since it concerns the ‘art and science’ of medicine on which the clinical decisions and judgments of the physician are made” [5]. For many years, most nephrologists identified kidney diseases by monocausal thinking and subsequently tried to categorize their findings in classifications of well-defined disease entities. In the last 20 years, pediatric nephrologists contributed substantially to the understanding of complexity in nephrology by identifying different genetic roots of kidney diseases with similar clinical symptoms. Their findings induced an increased splitting of diseases into subgroups that were previously thought to belong to the same entity. Neglecting the interactive role of multifactorial processes with the whole is, however, not without risk, as splitters may generate preoccupation with very small parts of diseases until the common whole is almost forgotten, such as for instance the individuality and complexity of the affected patients. Superimposing highly standardized treatment protocols on all patients bears the risk of losing individualized therapy out of sight. The above conflicts had already been identified by one of the pioneers in transplantation Roberto Burgio whose scientific research was based on in-depth work in the laboratory, but without ever loosing sight of the child [6].

How might systems thinking help pediatricians avoid reductive thinking and to improve a combination of deductive and inductive thinking in research? Edgar Morin wrote on “complexity and new science” [7]: “At the time of globalization, specialization drives the progress of knowledge; however, it also drives to breaking down knowledge which should be kept as a whole. The disjunction between disciplines hides the connections and the complexity of the whole human being. It is a paradox that medical progress induces regression of knowledge and causes new ignorance”. “We are in extreme need of transdisciplinary concepts to extract, assimilate and integrate knowledge which is broken down, separated, compartmentalized and fragmented.” “The management of societal problems will pose the most significant challenges” [8]. Physicians have learned from the Covid 19 pandemic that the purely medical is no longer in the foreground, but that it is about the interaction between virus and health authorities, between test strategies and bed capacities, and last but not least about the conflicts between people’s behaviour and medical and/or political measures.

## Complexity in pediatrics

**Fig. 1:**
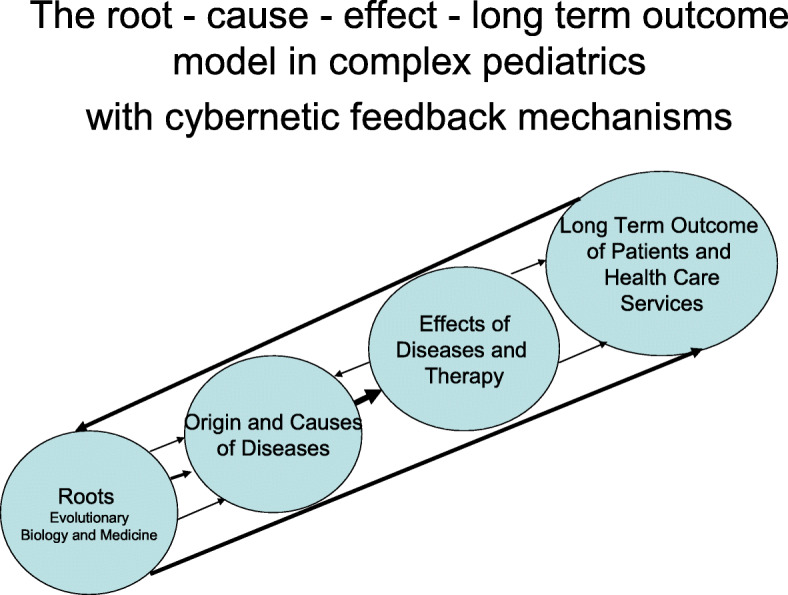
Short explanation of the cybernetic feedback mechanisms of roots, causes, effects and long term outcome of diseases from childhood to adult life

Evolutionary medicine uses complex systems thinking in pediatrics and means understanding the model of roots, causes, effects and long term outcome of diseases (Fig. 1). Evolutionary medicine is not a discipline like genetics [9]. It is an approach with which to analyse many different parts of medical science. Patients with acute onset of their disease may believe that a sudden illness came out of the blue, or they look for somebody or something they can blame for their disease. However, diseases may have their roots in the past of mankind and not only during fetal life or early childhood. Genetic abnormalities, incomplete fetal programming or early and symptom-free postnatal disturbances can be the roots for subsequent diseases, especially if a second hit occurs. The second hit may be falsely ascribed as the cause of a disease because it has a temporal association with the leading symptoms and signs of diseases. However, this view is too short-sighted. The diseases may turn out to be complex disorders and - after analyzing their etiology, pathology, clinical picture and responsiveness to treatment - they may no longer constitute the expected single entity.

Complex systems thinking in pediatrics means respecting the life cycle model. This means that each euro being invested in healthcare of young children will pay out several-fold in adult life [10]. Complex system thinking must include the process of setting priorities by pediatricians, other caregivers and opinion-makers. Priority in the context of child healthcare services means that each nation must define its own priorities and responsibilities when abolishing deplorable states of social affairs. Setting priorities means deciding on what must be done first and what can wait and when it is time to act. Priority-setting means answering the questions of where and how the experts can most effectively use their capacity and what the expected benefits will be.

What could the role of clinical philosophers be in pediatric care? The corona virus pandemics showed that better leadership in politics and medicine could help to cope with crises. Philosophers could explain the different types of leadership. Perhaps philosophers will tell pediatricians that it would be better to have collaborative than competitive leadership. Collaborative leaders will inspire communication, cooperation and consensus across internal and external borders. No national healthcare service system can reach high standards without cross-border care and international research, nor can national services in small countries cope alone with rare diseases.

## Complex system thinking in pediatrics

If our medical systems are excessively based on technology and belief in information, it is dangerous to perceive the child as an object of healthcare. Many national habits and rules were identified when comparing pediatric standard procedures among countries [11]. The large differences that existed in kidney healthcare services were not so much based on scientific findings but on national character [12]. Presidents of different national pediatric societies tended to assume that any deviation from what they perceived as the medical norm occurred only because other countries lacked the same financial resources, knowledge, organization or the will to do as they did [11]. Their view assumed that everyone in Europe was working towards the same pediatric goals, with some countries more successful than others. However, after many years of studying child healthcare services in Europe, Katz et al. [13] came to the conclusion that, even assuming unlimited financial resources for all European countries, their national goals might still not be the same because of different national priorities.

Fragmentation of child healthcare services demands a coordinating care team [14]. Evaluating diversity of child healthcare service systems in pediatrics means understanding national diversities and priorities [15]. Innovative ideas and progress are easier to grow and be implemented at the intersection of disciplines, rather than confined to a restricted space. The things that favor originality and innovation are international social responsibility, cross-border care and scope for academic freedom.

Could philosophy fill the gap that arises from deficits of the current healthcare service systems? Does it have to be a philosopher who becomes part of the pediatric team or should members of the team be trained by external philosophers in complex systems thinking? Teaching philosophy without training the whole team during practical work is unlikely to be the solution. It is also hard to believe that pediatric services could be analyzed and consulted philosophically from the outside. The improvement of healthcare systems first needs to clarify the current status, then to answer the question “What will happen in the future?”, and, finally, to define the urgency of the concern in order to eliminate deficits. Unfortunately, data on the role of philosophers in European pediatrics is lacking.

Is there a need for a survey of pediatricians relating to how they see the role of philosophy in pediatrics? Pediatricians look at healthcare services in a manner conditioned by their own culture. Learning across borders is essential for the training of young pediatricians. However, it is not easy to understand the influence of national behavior on healthcare if comparing one culture with another. Science progresses through the work of specialists who are mostly indispensable and may defend their individual originality in small niches as demonstrated by De Santo [16]. This knowledge points to the need of complex thinking. Thus, complexity is born by necessity. There are many problems to be solved in medicine which need more than medical interdisciplinarity. This is where philosophers may come in and join a medical team. One of the crucial questions is their integration into routine and busy clinical activities. Therefore, could a retired professor of pediatrics become the primary contact person for a practical philosopher? Both could transfer their knowledge and experience to younger pediatricians in a philosophical team, leading to an improved culture of communication, cooperation and consent. Moreover, as most retired professors have spent parts of their career in foreign countries, they know that there is no better way for pediatricians to understand cultural differences than studying and working abroad. Assuming unlimited financial resources of countries, the emeritus knows well that national goals might not be the same, because countries sometimes have different priorities [17]. As Aristotle said, “He who sees things from the beginning will have the best view”. Thus, there is certainly an influence of experience on deciding upon priorities. During the decision-making process the cognitive dissonance—which means a gap between conviction (I wish) and actions (I can)—must be taken into account, and the elderly generation may have developed a kind of wisdom or the obligation to be wise. More specifically, this could mean that philosophers and emeriti should aim at developing less passion, fewer emotions, less desire, fewer wishes for themselves. Experience and the use of the philosophical model of deconstruction will encourage a testing of the opposite extremes of conflicts, e.g. young and old members of staff, or nurses and physicians. Deconstruction means searching for a common denominator between young and old that is detectable and positive for both.

All these questions would arise in a situation in which a philosopher becomes an important member of a team of scientists and pediatricians, thus creating a cultural and scientific “parabiosis”.

## Philosophical practice

Shaped by the life skills of a medical community, philosophy can help to develop a special feeling for the ability of a whole society to endure what is unavailable in a health crisis. Philosophical practice can claim to treat sick societal behaviors. Anybody in a healthcare system could seek advice from philosophical practitioners, but in doing so be considered as a guest rather than patient or client. Philosophical practice is neither looking for the best of possible worlds, nor for easy solutions. Philosophy offers for caregivers in all disciplines a cognitive process which has no “blind spots”. Although unintended, complacency is inevitably increasing with time in people belonging to institutions such as health systems for longer periods. Philosophy consists in knowledge that abstains from “tunnel vision” and from inefficient communication. In philosophical practice, terms that may be qualified as pathological in the medical field are not excluded from discourses and are re-thought from the tradition of philosophy. Natural sciences are based on inductive thinking, which involves using the concept of a single fact that can explain the whole. The humanities and social sciences offer the use of deductive thinking, which means starting from a hypothesis and then singling out facts that can be extrapolated from the view of the whole in order to explain the individual parts. This pathway starts by grasping the idea from complex systems thinking and it is completed if the suggested single fact fits into the concept of the whole.

## What are the chances and pitfalls of a concept of proposing philosophers as a standby option in child healthcare services?

In principal, philosophy cannot offer solutions which medical experts fail to solve. Instead, philosophy offers new pathways of thinking. Philosophers should not become a member of internal hierarchies and philosophers should not become the referees for internal conflicts. The financing of their job is a challenge and must be regulated internally. One of the main problems could arise from the dearth of adequately trained clinical philosophers.

## Conclusions

We suggest that pediatrics should adopt the method of complexity and explore the zone of contact with philosophy. The latter link might represent a strategic tool in educating a cadre of Renaissance scholars, like those who accomplished the peak achievements of Florence at the time of Medici in the fourteenth century [6]. We conclude that studying the associations of theory and practice in complex health systems—like pediatrics—in countries with different historical and political bases requires methods which differ partially from natural sciences. What pediatricians are, depends on where and when they live. Moreover, children cannot live healthily in a sick country. Pediatricians cannot work efficiently unless decision-making processes in research and care are supported by academic freedom, complex systems thinking, philosophy and bioethics. Being in awe of the child’s life must always be at the center of pediatric action.

## Data Availability

Not applicable.
